# Fatty Acid Composition and Metabolism in *Leishmania* Parasite Species: Potential Biomarkers or Drug Targets for Leishmaniasis?

**DOI:** 10.3390/ijms24054702

**Published:** 2023-02-28

**Authors:** Marine Leroux, Céline Luquain-Costaz, Philippe Lawton, Samira Azzouz-Maache, Isabelle Delton

**Affiliations:** 1 CNRS 5007, LAGEPP, Université of Lyon, Université Claude Bernard Lyon 1, 69100 Villeurbanne, France; 2 Department of Biosciences, INSA Lyon, 69100 Villeurbanne, France

**Keywords:** fatty acids, lipid mediators, trypanosomatids, leishmania, leishmania/macrophage interactions

## Abstract

Fatty acids have received growing interest in *Leishmania* biology with the characterization of the enzymes allowing the complete fatty acid synthesis of this trypanosomatid parasite. This review presents a comparative analysis of the fatty acid profiles of the major classes of lipids and phospholipids in different species of *Leishmania* with cutaneous or visceral tropism. Specificities relating to the parasite forms, resistance to antileishmanial drugs, and host/parasite interactions are described as well as comparisons with other trypanosomatids. Emphasis is placed on polyunsaturated fatty acids and their metabolic and functional specificities, in particular, their conversion into oxygenated metabolites that are inflammatory mediators able to modulate metacyclogenesis and parasite infectivity. The impact of lipid status on the development of leishmaniasis and the potential of fatty acids as therapeutic targets or candidates for nutritional interventions are discussed.

## 1. Introduction

Leishmaniases are a complex of tropical and subtropical diseases caused by unicellular parasites of the genus *Leishmania* transmitted by a sandfly vector. The localization of parasites either in dermal macrophages or their migration to different tissues within internal macrophages contribute to disease establishment in the host, causing different phenotypes such as cutaneous (CL), mucocutaneous (MCL), and visceral leishmaniasis (VL) [1,2,3]. Twenty *Leishmania* species are distributed worldwide and transmitted to humans and animals by the sandfly under the different clinical forms mentioned above. Nearly 350 million people are exposed to leishmaniases in more than 90 countries worldwide, mainly in India and Africa. The emergence of leishmaniases is now observed throughout the Maghreb, the Middle East, and South America, with around 2 million new cases reported annually [4]. Canine leishmaniasis is endemic in more than 70 countries in southern Europe, Africa, Asia, and South and Central America and it has also been reported in the United States of America [5]. Dogs constitute a major reservoir for the *Leishmania* species that also affect humans, and several hundred cases occur annually in humans in the Mediterranean basin (WHO, 2020).

The *Leishmania* life cycle involves two different stages: the promastigote inside the insect vector and the amastigote in the vertebrate’s macrophages. The promastigotes inoculated by the vector are internalized by host macrophages via phagocytosis and undergo a transformation into the amastigote stage within a parasitophorous vacuole of phagolysosomal origin [6]. Differentiation of promastigotes into amastigotes involves complex mechanisms including morphological changes and genomic expressions such as surface molecules that are required for parasite infectivity and survival into macrophages. Entry of intracellular pathogens and cell differentiation are generally associated with the reorganization of the plasma membrane where lipids, essentially phospholipids, glycolipids, and sterols, are the main components. The host cell’s plasma membrane serves as a portal for the entry of intracellular pathogens [7]. There are also intricate relationships between parasites and host cell intracellular organelles through interactions with the parasitophorous vacuole membranes [7,8,9].

During the last decades, advances in biochemical and molecular approaches have both contributed to highlighting the importance of lipids and lipid metabolism in *Leishmania* biology and the course of macrophage infection. Highly sensitive analytical techniques such as liquid chromatography–mass spectrometry (LC-MS/MS) allow the revealing of lipidomic signatures related to the *Leishmania* life cycle [10,11] and lipid alterations in drug-resistant *Leishmania* strains [12,13]. Genomic and transcriptomic approaches have also reported some correlations between lipid-related gene expression and antileishmanial drug resistance [14,15]. To date, fatty acids (FA), phospholipids (PL), and sterols have emerged as biological actors in *Leishmania* physiology and virulence [12,13].

Recent reviews have emphasized the potential of enzymes involved in lipid and FA synthesis and metabolism to serve as targets for anti-trypanosomal drugs [12,16]. In this review, we will focus on the specificities of *Leishmania* parasites in terms of FA composition and the production of oxygenated FA derivatives in both promastigote and amastigote forms and in interaction with host cell macrophages. We will open an opinion on the potential of FA to reveal biomarkers for leishmaniasis and to emerge as therapeutic targets or as candidates for nutritional intervention.

## 2. *Leishmania* Species and Leishmaniasis

The diversity of *Leishmania* species and their wide geographical distribution as well as their sensitivity to treatments illustrate the influence of the environment and the microenvironment, whether of the host or reservoirs, on the development of the disease and species tropism.

The reference method for *Leishmania* typing is multilocus enzyme electrophoresis (MLEE). The most commonly used is the Montpellier system (MON) which is based on the analysis of 15 enzymes. As an example, *L. infantum* is characterized by a large enzymatic polymorphism and comprises 30 zymodemes in humans. Some of them are related to VL (MON27, 28, 72, 77, and 187), others only to CL (MON11, 29, 33, 78, and 111), and some zymodemes can cause both VL and CL, such as MON1 and MON24 [17,18,19,20].

*Leishmania* species cause different clinical forms depending on the localization of the parasite in the host. VL or Kala Azar in the Indian subcontinent is the most serious form and its evolution is fatal in the absence of treatment. It is caused by two species: *L. donovani*, highly endemic in East Africa and the Indian subcontinent, anthroponotic and affecting all age groups; *L. infantum*, zoonotic with a canine reservoir, located in countries around the Mediterranean, the Middle East, and South America, pathogenic in children and immunocompromised (HIV) patients [4,21]. CL is the least severe form of the disease. It is caused by different *Leishmania* species such as *L. major* and *L. tropica* in the old world, *L. amazonensis*, *L. guyanensis*, *L. panamensis,* and *L. braziliensis* in various regions of Central and South America [22,23]. Diffuse CL is a much rarer form linked to immunosuppression and is more difficult to treat. In the old world, it is caused by *L. aethiopica*, while in the new world, it is caused by *L. mexicana* and *L. amazonensis* [24,25]. MCL or “espundia” appears from a few days to several years after CL. It is caused by *L. braziliensis*, *L. panamensis*, *L. amazonensis,* and *L. guyanensis* [26]. South America is the most important endemic area. Post-kala-azar leishmaniasis (PKDL) is a nonfatal dermatological complication that occurs in some patients with VL [27]. It is still not understood why some species of *Leishmania* cause cutaneous symptoms while other species, which are fatal for humans and animals, cause visceral symptoms. Even more challenging is the double tropism of *L. infantum* or the shifting tropism from CL to VL in Leishmania/HIV co-infections [17]. It has been globally proposed that the development of the disease depends mainly on the intrinsic metabolism of the parasite species, although factors in the vectors and the biological status of the host have also been involved [28].

## 3. FA Profiles of *Leishmania* Lipids

### 3.1. FA Profile and Distribution in Lipids, Phospholipids, and Phospholipid Classes

Fatty acid compositions of total lipids and major lipid classes (e.g., total PL, triglycerides TG, unesterified FA) have been investigated in several species of *Leishmania*, especially *L. infantum, L. donovani, and L. major*. Overall, FA composition was conserved regardless of the lipid class or these *Leishmania* species, with a predominance of C16 and C18 FA [29,30,31,32,33,34,35,36]. These include the saturated FA palmitic acid (PA, 16:0) and stearic acid (SA, 18:0), the monounsaturated oleic acid (OA, 18:1n-9) and the polyunsaturated fatty acids (PUFAs) essentially linoleic (LA, 18:2n-6), and to a lower extent, alpha-linolenic (ALA, 18:3n-3) and gamma-linolenic acid (GLA, 18:3n-6). Longer chain FAs were mainly represented by elevated levels of n-3 PUFA such as docosahexaenoic (DHA, 22:6n-3) and eicosapentaenoic acid (EPA, 22:5n-3) in contrast to n-6 PUFA such as arachidonic acid (AA, 20:4n-6) and docosapentaenoic acid (DPA, 22:5n-6) that are present at only trace amounts [32,34,35,36]. DHA was recovered in the three lipid classes; however, its proportion was the highest in TG reaching up to 5% of total FA [35,36]. The predominance of C16 and C18 acyl chains was recovered in TG [32]. Lipid analyses have also revealed the presence of one single major cyclopropanated FA (CFA), cis-9,10-methyleneoctadecanoic acid (C19Δ, dihydrosterculic acid), in several *Leishmania* species such as *L. donovani, L. infantum, L. braziliensis* but not *L. major or L. tropica* [37,38,39]. C19Δ constitutes a minor component representing less than 1% of the total FA content.

Some differences in FA distribution are also remarkable among individual PL (e.g., phosphatidylcholine PC, phosphatidylethanolamine PE, phosphatidylinositol PI, and sphingomyelin). In *L. donovani* promastigotes, LA was the most abundant FA in PE with only trace amounts of higher PUFAs; OA and ALA were major acyl chains in PC and PI, respectively [29,31]. Longer chain PUFAs such as DHA and AA were found in high proportions in diphosphatidylglycerol [29], PC, and PI [30]. Analyses of molecular species showed the predominance in *L. donovani* of C16 and C18 acyl chains in PC, PE, and PI, as well as DHA-containing species in PC [32]. Wassef et al. [30] showed that the relative distributions of FA in PC, PE, and PI were different between PL isolated from whole cells and those isolated from surface membranes, with a higher ratio of unsaturated to saturated FA in PL surface membranes. In *L. mexicana* and *L. infantum*, C19Δ was mainly recovered in plasmenylethanolamine, the dominant class of PE in *Leishmania* [37,38,39].

### 3.2. Interspecies Differences and Similarities in FA Composition

In addition to *L. infantum*, *L. donovani,* and *L. major*, as summarized above, FA profiles were determined in many other *Leishmania* species including *L. tropica*, *L. mexicana*, *L. amazonensis,* and *L. tarentolae* [29,30,31,32,33,35]. Using nonmetric multidimensional scaling analysis, de Azevedo et al. showed that *L. infantum/chagasi* and *L. amazonensis* present different FA profiles; however, the method used did not allow the identification of which FA. GCMS analysis indicated that DHA was not detected in these two species, unlike other *Leishmania* species [33].

To further investigate interspecies specificities, we have compared the FA composition of total lipids from nine *Leishmania* isolates in the same series of analyses to limit methodology variability. Isolates were of human or canine origin, causing either visceral (*L. infantum*) or cutaneous (*L. tropica*, *L. major,* and *L. infantum*) leishmaniasis in Tunisia (Table 1). The data indicate only small differences between the *L. infantum* and *L. tropica* strains, regardless of the human/canine origin or the visceral/cutaneous form. These strains all contained about 30% saturated fatty acids (SFA), 20% monounsaturated fatty acids (MUFAs), and 50% polyunsaturated fatty acids (PUFAs), with quite an even distribution between the n-6 and the n-3 series. Within each fatty acid series, the C18 fatty acids were found in the highest proportions, consistent with previous analyses on total lipids or lipid/PL classes of various *Leishmania* species [29,30,31,32,33,35]. Long-chain n-6 PUFAs were present in a much lower proportion than the long-chain n-3 PUFA, especially AA, which accounted for 1% of total FA compared to 10% for DHA. While the FA profiles of *L. infantum* and *L. tropica* are broadly similar, some differences are noticeable in the *L. major* strains, such as a significantly higher proportion of myristic acid (14:0) and lower proportions of n-3 FA, especially ALA and DHA. A recent study reported the FA composition of *L. major* strains of different host origins—human or rodents by combining in silico and GC–MS [36]. The authors also pointed out some differences in *L. major* compared to other *Leishmania* species, notably the absence of detectable ALA and DPA. From our observations and [36], *L. major* is the species whose FA composition shows the most differences (high 14:0; low ALA and DHA) compared to other *Leishmania* species. Of interest, 14:0 and DHA are among biologically active FAs in *Leishmania* pathogenicity (see paragraph 4) and ALA is an essential precursor for n-3 PUFA including DHA. Moreover, although the FA composition of *L. major* strains of human and rodent origin is globally conserved, the rodent clones show a lower quantitative abundance of LA, which is correlated with lower infectivity against macrophages [36]. Whether or not specific FAs turn out to be species biomarkers and the differences in FA composition contribute or not to parasite pathogenicity deserves further investigation.

### 3.3. Changes in FA Composition Relating to Parasite Differentiation and Drug Resistance/Sensitivity

Some attempts have also been made to identify specific FAs as biomarkers of biological processes in *Leishmania* parasites.

In this respect, we showed that the FA composition of *L. donovani* and *L. infantum* lipids changed during the differentiation of the promastigote form into the amastigote form inside the host macrophages [35]. The most pronounced changes in amastigotes were the increase in total n-3 FA especially DHA in total PL, and the decrease in total n-6 FAs in all lipid classes analyzed (total PL, TG, and free FA). Noteworthily, there was a remodeling in the distribution of n-6 FA in PL with a significant increase in AA at the expense of its precursors LA and GLA which were conversely reduced. Since the FA composition of amastigotes was close to those of host cells, we have suggested that the remodeling of FAs in amastigote lipids could either depend on FA and/or desaturase/elongase activities available in macrophages or the regulation of FA biosynthesis enzymes in amastigotes.

Changes in FA compositions have been observed in several *Leishmania* species upon exposure to antileishmanial drugs as well as in resistant *Leishmania* strains. These changes have been proposed as putative mechanisms for drug toxicity or the development of drug resistance.

It is assumed that the unsaturation level of plasma membrane PL modulates membrane fluidity, which could impact drug membrane interactions and/or drug transport. A diminution of membrane fluidity has been reported in various *Leishmania* parasites resistant to antileishmanial drugs such as miltefosine [40], amphotericin B [41], and antimony [33,42]. In Rakotomanga et al. [40], the authors described the FA changes in miltefosine-resistant *L. donovani* strains. They observed a significant decrease in unsaturated alkyl chains in PL of miltefosine-resistant parasites, such as OA, AA, and ALA chains, that could result from reduced desaturation activities. It was proposed that the lower PL unsaturation in miltefosine-resistant parasites would reduce membrane fluidity and impair miltefosine affinity [43]. Decreased levels of unsaturated FAs were also reported in *L. donovani* strains resistant to amphotericin B compared to sensitive strains, nevertheless, the increase in membrane fluidity was rather attributed to sterol changes [41]. By contrast, FA profiles of *L. donovani*, *L. chagasi,* or *L. amazonensis* strains resistant to antimony revealed an increased level of unsaturation compared to sensitive strains, which was hypothesized to impair antimony transport and therefore antimony sensitivity [44,45]. Altogether, although correlations between unsaturation level and drug resistance have been reported, the data are variable depending on *Leishmania* strains and/or drugs.

Several studies have pointed out variations in some fatty acids associated with drug resistance. Exposure of *L. donovani* parasites to antimony induced a marked downregulation of OA and conversely a marked upregulation of vaccenic acid (18:1n-7) and very long-chain FAs including AA and DHA [34]. These very long-chain FAs would first be responsible for antimony-induced killing through the generation of oxidative stress in treated parasites, and secondarily causative of antimony resistance by increasing ergosterol synthesis. The resistance of *L. infantum/chagasi* and *L. amazonensis* isolates to antimony was also associated with a marked decrease in OA while AA showed the opposite trend [33,42]. Bouadid et al. [36] showed that the rodent clone of *L. major* with the lowest amount of LA was the least sensitive to miltefosine.

More extensive lipidomic studies have also highlighted specific changes in lipid species that could turn out to be biomarkers of drug-sensitive vs. drug-resistant *Leishmania* parasites. These include PE species (C19Δ or 24:0-enriched species) and inositolphosphoceramide (IPC) species in *L. infantum* or *L. donovani* strains resistant to miltefosine or amphotericin B [44,45]. Gutierrez Guarnizo et al. [46] have shown that *L. tropica* strains resistant to antimony exhibited a strong downregulation of PC, especially 16:1, 18:2, or 18:3-containing species, while sensitive strains strongly upregulated TG with long-chain FAs after drug exposure.

## 4. FA Acquisition in *Leishmania*

It is commonly admitted that *Leishmania* parasites acquire FAs both by de novo synthesis and by the uptake of lipids from their host environment [47].

### 4.1. De Novo Synthesis of FA and PUFA in Leishmania Parasites

The pathways of de novo FA synthesis in *Leishmania* have been extensively described in recent reviews [16,48] and will only be briefly presented here. It should be noted that comparative studies have been carried out on other trypanosomatids highlighting some specificities for *Leishmania*. Most of the available data come from genomic and biochemical studies in *L. major*, *T. cruzi*, and *T. brucei*. Several enzymes involved in de novo FA synthesis have been characterized in trypanosomatids and have emerged as potential targets for antileishmanial drugs [16].

*Trypanosoma* and *Leishmania* use a mitochondrial type II FA synthesis pathway (FAS II) that mediates the synthesis of caprylate (C8) and palmitate (C16). Three genes coding for at least two of the four enzymes involved in the FAS II pathway have been identified in *L. major*. Trypanosomatids also use the unconventional elongase (ELO) system to synthesize FAs [47,49]. ELO enzymes are integral membrane proteins of the endoplasmic reticulum that catalyze the extension of acyl chains. Genomes of *L. major* and *T. brucei* have genes encoding putative ELO1-3 proteins to synthesize FAs from C4 to C18. ELO1 extends C4 to C10, ELO2 extends C10 to C14, and ELO3 extends C14 to C18. Additional ELOs such as ELO4, ELO5, and ELO6 are involved in the extension of PUFAs [47,49].

Desaturases are enzymes responsible for the synthesis of unsaturated FAs and PUFAs by the insertion of double bonds in the FA carbon skeleton. Several desaturases including Δ4, Δ5, Δ6, Δ9, Δ12, and Δ15 were characterized in *L. major*; only Δ4, Δ9, and Δ12 desaturases were identified in *T. brucei* and *T. cruzi* [50]. Of note, as Δ12 desaturase activity is not detected in mammals, this enzyme turned out to be a potential drug target for novel therapeutics against trypanosomatids.

The first step common to the three parasites involves a desaturase Δ9FAD (stearyl-Coenzyme A desaturase) which converts stearic acid 18:0 (formed de novo or captured in the host) into 18:1n-9. *Leishmania* is able to synthesize 18:2n-6 and 18:3n-3 from 18:1n-9 using the enzyme ∆12Des [49]. A particularity of *L. major* is to express the enzyme ∆15FAD which allows the conversion of 18:2n-6 to 18:3n-3. In this parasite, a series of reactions makes it possible to convert 18:2n-6 into 20:4n-6 and 18:3n-3 into 20:5n-3 according to successive stages of elongation and desaturation, which use desaturases (∆6 and ∆5FAD) as well as an elongase (∆6ELO). ∆5ELO converts 20:4n-6 to 22:4n-6 as well as 20:5n-3 to 22:5n-3; ∆4FAD allows the synthesis of 22:5n-6 and 22:6n-3. The enzymes ∆5ELO and ∆4FAD are also present in *T. brucei* and *T. cruzi* [47]. *Leishmania*, therefore, have three additional desaturases which are absent in *T. brucei* and *T. cruzi*, Δ15 FAD, Δ6, and Δ5 FAD as well as the elongase Δ6ELO.

Cyclopropanated fatty acids (CFAs) are generated from cyclopropane fatty acid synthase (CFAS) that catalyzes the transfer of a methylene group from S-adenosyl methionine to an unsaturated FA. The gene encoding CFAS has been identified in several *Leishmania* species. Including *L. infantum, L. donovani, L. mexicana,* and *L. braziliensis,* but is missing in *L. major* [37]. CFAS has been characterized in both promastigote and amastigote forms of the parasite in *L. infantum* and *L. mexicana* [37].

### 4.2. FA Uptake

Several studies have suggested the presence of FA binding proteins (FABP) and FA transport protein (FATP) that would be involved in the specific uptake of FAs in leishmanial parasites [51]. Orthologues of human FATP have been identified in the genome of *L. major* [47]. The uptake of free FAs (PA, SA, and OA) and their esterification in glycerolipids has been described in axenic amastigotes from *L. mexicana* [52]. We recently showed that supplementation of the culture medium of *L. infantum* promastigotes with AA or DHA led to specific FA enrichment in parasite lipids indicating that promastigotes efficiently uptake exogenous FAs [53]. Specific FA enrichment of promastigote lipids was also observed after supplementation with OA (unpublished observations).

Plasma lipoproteins have been described as an important source of lipids, especially cholesterol for trypanosomatids, through internalization processes [54,55,56]. Low-density lipoprotein (LDL) is likely a source of FAs as it contains large amounts of FAs esterified in TG and cholesterol esters.

## 5. Significance of FAs in Parasite/Host Interactions and Parasite Survival in Host Cell

Several studies have investigated the role of some specific FAs in *Leishmania* parasites at both promastigote and amastigote stages, through the exogenous supply or inhibition of their biosynthesis. Among them, 14:0, some PUFAs and cyclopropanated FAs have emerged as essential or potential actors in *Leishmania* biology.

### 5.1. Myristic Acid (14:0) and Myristoylation

Myristic acid (14:0) is important for N-myristoylation which consists of the transfer of C14 from myristoyl-CoA onto the N terminal glycine residue of cellular proteins. This transfer is catalyzed by the enzyme N-myristoyltransferase (NMT) which has emerged as a potentially druggable enzyme in *Leishmania* [57]. NMT has been characterized in *L. donovani* and *L. major* and reported for both *Leishmania* species to be essential for cellular growth, vesicular trafficking, and survival in the mammalian host [57,58,59]. Thirty high-confidence N-myristoylated proteins have been identified with roles in protein phosphorylation, protein transport, and degradation and Golgi functions in both promastigote and amastigote stages of *L. donovani* [60].

The essentiality of NMT In *Leishmania* viability has been demonstrated by both genetic and pharmacological approaches. Double knockout is lethal in *L. donovani* and *L. major* promastigotes [57,58]. In vivo studies using the plasmid shuffle method further demonstrated that NMT is also essential for the viability of intracellular amastigotes of *L. donovani* [61]. Several pharmacological NMT inhibitors have been developed that exert killing activity on *Leishmania* promastigotes although failing to inhibit axenic or intracellular amastigotes or show low selectivity over human NMT [57,62]. Despite low activity toward *L. donovani* amastigotes, Corpas-Lopez et al. have shown that the pharmacological inhibition of NMT significantly reduced parasite burden in a mouse VL model, therefore, validating NMT as a pharmacological target in *Leishmania* [63].

Myristate is also a component of glycosylphosphatidylinositol (GPI) lipid anchors that attach major classes of surface molecules such as promastigote surface protease (or gp123) to the plasma membrane of the *Leishmania* promastigote. These surface proteins with GPI anchors play a crucial role in *Leishmania* recognition [59].

### 5.2. Cyclopropanated Fatty Acids

The functions of CFA and CFAS are not totally understood and vary depending on the *Leishmania* species. Subcellular fractionation studies indicate that the cyclopropanated FA C19Δ mainly locates in both the endoplasmic reticulum and plasma membrane-enriched fractions in *L. infantum* [37]. CFAS was preferentially detected during the log and early stationary phases of promastigotes in *L. infantum* and *L.mexicana* promastigotes and in *L. infantum* amastigotes upon macrophage infection [37,39]. In *L. infantum* promastigotes, knockout of the CFAS gene lowered parasite burdens in the spleen and liver during in vivo mice infection [37]. In *L. mexicana*, CFA modifies the fatty acid chain of plasmenylethanolamine [39]. CFAS plays a key role in the regulation of the cellular shape of *L.mexicana*, its resistance to acidic environments, and to cell membrane targeting of lipophosphoglycan, but in contrast to *L. infantum*, is not essential for parasite virulence [39]. Increased content of C19Δ has been reported in resistance to antileishmanial drugs such as amphotericin B and miltefosine supporting its role for the pathogenicity or survival of the parasite [44].

### 5.3. PUFAs: LA, AA and DHA

PUFAs such as LA, AA, and DHA are known to play a key role in maintaining membrane fluidity and regulating inflammatory and oxidative status. Whether they could have an impact on parasite infectivity and survival is, therefore, an interesting but still understudied issue.

Saini and Rai [64] showed that LA supplementation of culture medium during macrophage infection with *L. donovani* promastigotes decreased parasite load by strengthening macrophage inflammatory response. LA supplementation also inhibited *L. donovani* promastigotes from secreting exosomes containing immunomodulatory factors.

Recently, by carrying out AA supplementation on two *L. infantum* strains, a visceral MON-1, and a cutaneous MON-24, we showed that promastigote lipids became enriched with AA, which correlated with higher infectivity toward J774 macrophages. DHA supplementation induced DHA enrichment of lipids which was also associated with higher infectivity, although specifically for MON-24 and not the MON-1 strain [53]. We proposed that these effects could be mediated through the accumulation in supplemented promastigotes of PUFA-derived oxygenated metabolites exhibiting pro/anti-inflammatory activities (see paragraph 5).

Due to their high level of unsaturation, AA and DHA are very sensitive to peroxidation, thus promoting the production of reactive oxygen species (ROS) [65]. Likewise, exogenously added AA and DHA were shown to promote ROS production in *L. donovani* promastigotes [34]. Several studies have reported an increase in ROS production in either murine or human macrophages during *Leishmania* (*L. chagasi, L. amazonensis, and L. braziliensis*) infection, most likely as a defense mechanism to eliminate parasites by activating inflammatory and immune signaling pathways [66,67,68,69,70]. In our AA and DHA supplementation studies, we found that ROS production induced in macrophages upon *L. infantum* infection was similar for control and supplemented parasites [53]. However, since parameters other than ROS production (including antioxidant enzyme activity and vitamin E level) contribute to the fine regulation of oxidative status, we cannot exclude that infection with promastigotes supplemented with AA or DHA may actually increase oxidative stress in macrophages.

Furthermore, it was recently shown that PUFAs including AA, EPA, and DHA stimulate the formation of lipid bodies (LB) in *L. braziliensis* and *L. infantum* procyclic promastigotes [71]. As mentioned below, LB has recently emerged as an important organelle in *Leishmania* for lipid metabolism and parasite pathogenicity.

## 6. PUFA Oxygenated Metabolism

Once released from membranes via phospholipases, PUFAs are precursors of various active oxygenated metabolites, also called oxylipins, such as eicosanoids derived from AA or EPA, and docosanoids derived from DHA. These lipid mediators are formed via the activation of pathways involving dioxygen-dependent oxidation, either enzymatically-dependent using cyclooxygenase (COX), prostaglandin synthase (PGS), lipoxygenase (LOX), or cytochrome P450 oxygenases (CYP), or nonenzymatic through a free radical reaction under oxidative stress conditions. Oxylipins gather prostaglandins (PG), leukotrienes (LT), hydroxy-eicosapentaenoic (HEPE), hydroxy-eicosatetraenoic (HETE), hydroxy-docosahexaenoic (HDoHE), epoxy-eicosatrienoic (EET), and oxo-eicosatetraenoic (oxo-ETE) acids as well as proresolving mediators (resolvins, maresins, and protectins). Oxygenated metabolites regulate various biological processes including inflammation, blood coagulation, neuroprotection, and pain response. It is well established that AA is the precursor of proinflammatory mediators, while DHA is conversely converted into anti-inflammatory derivatives—so-called proresolving mediators [72,73]. Nonenzymatic cyclic oxygenated metabolites, known as isoprostanes and neuroprostanes are mainly used as biomarkers of oxidative stress [74].

Eicosanoids play an important role in *Leishmania* infection, as parasite infection results in an intense inflammatory response into host cells (macrophages) associated with an increased expression and release of proinflammatory mediators. The balance between lipid mediators, especially leukotriene B4 (LTB4) issued from 5-LOX and prostaglandin E2 (PGE2) issued from PGE2 synthase (PGE2S), determines the macrophage inflammatory response and the parasite survival. The activity of 5-LOX helps macrophages in eliminating parasite infections such as *T. cruzi* [75,76], *L. donovani, L. amazonensis* [77], and *L. infantum* [78]. *L. major*-infected neutrophils release large amounts of LTB4 during the first hours of infection [79]. LTB4 is involved in NO production and reduces parasite load in different cellular models of *L. amazonensis* infection [80,81,82,83,84]. With respect to PGE2, its production is induced in macrophages infected with *L. infantum and L. donovani* [85,86], *L. amazonensis*, and *L. major* [86] and benefits parasite survival [87]. Indeed, PGE2 promotes the growth of *L. major* [88] and *L. donovani* [77] and reduces the macrophage immune response against *L. donovani* [86]. In mice infected with *L. mexicana* and treated with a COX inhibitor, a reduction in lesion size was observed associated with reduced levels of PGE2 in splenocyte supernatants [89]. However, PGE2 was conversely reported to exert antileishmanial activity. It induced *L. amazonensis* or *L. infantum* killing in infected macrophages [86,87]. PGE2 released by macrophages under exposure to *L.infantum* was shown to mediate a proinflammatory response [90]. PGE2S was also highly upregulated in macrophages infected with *L. donovani* [91].

**Figure 1:**
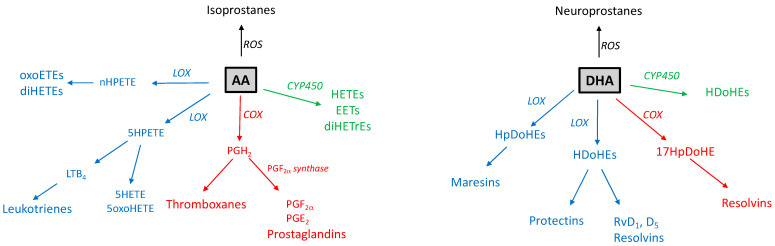
The biosynthesis of AA- and DHA-derived active lipids in trypanosomatids. LOX enzymes are in blue, COX enzymes in red, and CYP450 enzymes in green. Arachidonic acid (AA) can be metabolized into thromboxanes and prostaglandins by the COX pathway; into hydroxyeicosatetraenoic acid (HETEs), leukotrienes, oxoeicosatrienoic acids (oxoETES), dihydroxyeicosatetraenoic acid (diHETEs) via the LOX pathway; into HETEs, epoxyeicosatrienoic acids (EETs), dihydroxyeicosatrienoic acid (diHETrEs) via CYP450. Docosahexaenoic acid (DHA) can be metabolized into hydroxydocosahexaenoic acids (HDoHEs), D-resolvins (RvD1; RvD2), maresins, protectins via the LOX pathway, and hydroxydocosahexaenoic acid (HDoHEs) via the CYP pathway. 17-hydroperoxydocosahexaenoic acid (17-H(p)DoHE) issued from the COX pathway is the precursor of DHA-derived specialized proresolving mediators.

Besides the production of lipid mediators by infected macrophages, trypanosomatid parasites have also been shown to produce both proinflammatory and proresolving mediators, thereby modulating macrophage responses to parasite infection [71,87,92,93,94,95] (Figure 1). These parasites possess the same classes of eicosanoids biosynthesis enzymes as mammals, including COX, LOX, and cytochrome P450 (CYP450) as well as parasite-specific enzymes [96]. Many parasites modulate host immune response through PG, but only a few COX activities have been described in trypanosomatids. PGF2α synthase (PGF2S) was identified in *T. brucei* [97] as well as in *T. cruzi* modulating the parasite infection [98]. PGF2S expression is increased during metacyclogenesis in *L. infantum, L. braziliensis, and L. amazonensis* [71]. In *L. infantum/chagasi,* the production of PGF2S is carried out in the lipid bodies [99]. The overexpression of PGF2S in *L. braziliensis* increased parasite virulence [100]. The metalloprotease gp63 was identified *in L. mexicana* as being responsible for COX2 activity in both promastigote and amastigote forms [101]. Several PGs, including PGE2, PGD2, and PGF2α, are produced by trypanosomatid parasites among which PGF2α is the dominant eicosanoid species in *L. donovani, L. tropica, L. major,* and *T. brucei* [102,103]. PGF2α produced by *L. infantum* is important for parasite virulence and increases the parasite burden [99].

Resolution of inflammation is an active process that promotes the normal function of infected tissues. Among proresolving mediators, resolvin D1 (RvD1) protects inflammatory responses and restores tissue homeostasis [103]. RvD1 administration is beneficial in *Leishmania* infection in the modulation of the cutaneous manifestation of the disease [87]. During *T. cruzi* infection, the parasite itself produces proresolving lipids such as RvD1, RvD5, and RvE2 associated with a modulation of host cell responses to infection [92]. RvD1 also favors *L. amazonensis* infection by promoting intracellular parasite replication in human macrophages [104]. Recently, Paloque and co-workers [94] reported the production of PUFA-derived oxygenated metabolites in *L. infantum*. They compared proinflammatory and proresolving mediator profiles in noninfectious (procyclic) and infectious (metacyclic) *Leishmania* promastigotes, revealing that oxygenated metabolites of AA and DHA were increased in the metacyclic form, partially depending on CYP-like enzyme activities. The highest increases were shown for 5- and 8-HETE derived from AA, and 14- and 17-HDoHE derived from DHA. HdoHE are the precursors for resolvins and maresins and the authors further showed that lipid extracts of infectious promastigotes induced the production of RvD2, maresin1, and protectin Dx in host macrophages and their polarization into the M2 phenotype. We recently showed that the *L. infantum* promastigote was able to convert exogenous AA and DHA into active metabolites, leading to the accumulation of EET, 5- and 8- HETE, and 14- and 17-HDoHE associated with an increase in parasite infectivity [53]. Another recent study quantified the production of eicosanoids in different *Leishmania* species including *L. infantum*, *L. amazonensis*, and *L. braziliensis* in the presence of exogenous AA. Several compounds were identified, including PGE2, PGD2, PGF2α, and numerous HETEs [71].

An alternative mechanism for the generation of prostaglandin-like compounds is the nonenzymatic generation of molecules called isoprostanes. Increased oxidative stress associated with increased levels of isoprostanes was measured in sera from patients suffering from CL [105]. Specific aldo-keto reductase (AKR) was identified in *T. cruzi and L. donovani*, converting AA into isoprostanes, especially 8isoPGF2α [106].

## 7. Therapeutics Insights: Lipids and Fatty Acids as Druggable Targets in Leishmaniasis

### 7.1. Current Treatments

The treatment of leishmaniasis in its various forms is limited to a restricted number of molecules, most of which are toxic; their administration is difficult and parasite resistance has developed. Several current therapeutics have been shown to target lipid metabolism, as recently reviewed by Arya et al. [16]. The major treatments and their interaction with lipid metabolism are only briefly described below.

Pentavalent antimonials (SbV) are the first-line drugs used to treat visceral, cutaneous, and mucocutaneous leishmaniasis. The mechanism of action of SbV and its active trivalent form (SbIII) is not completely established but it may interfere with oxidative status and fatty acid oxidation [52,107]. Antimonials are administered intralesionally or parenterally and produce severe side effects, such as cardiotoxicity, nephrotoxicity, and hepatotoxicity. These compounds have developed serious resistance in certain endemic areas, such as in northern Bihar in India [108]. In the new world, mainly in Brazil, the effectiveness of these drugs is over 90% while in Bihar, India, and Nepal, the treatment failure is around 60%.

Amphotericin B, the second treatment against leishmaniasis, is a polyene antibiotic isolated from *Streptomyces nodosus* first used as an antifungal in the treatment of systemic mycoses. It is currently used by parenteral administration against severe forms including VL or forms resistant to antimonials [109]. Amphotericin B acts on both promastigotes and amastigotes by targeting ergosterol in the parasite surface membrane and increasing permeability [22]. It also stimulates the phagocytic capacities of macrophages. In the new world, mainly Brazil, Amphotericin B has been used successfully to treat VL in special conditions, including older patients, children, transplant recipients, and patients with comorbidities such as diabetes and HIV infection, but it presents strong renal and hematological toxicity. Liposomal amphotericin B is significantly less nephrotoxic while remaining very effective and is at present the treatment of choice for immunocompetent patients in the Mediterranean region and the preferred drug for HIV/visceral leishmaniasis co-infection. The main obstacle to its widespread use, especially in the world’s poorest countries, is its high cost.

Miltefosine is an alkyl-lysophospholipid originally developed for the treatment of cancer that exerts antileishmanial activity by inhibiting the biosynthesis of *Leishmania* phospholipids and sterols [110]. It is the first oral drug available for the treatment of VL and CL and shows 95% efficacy against moderate VL in the Indian subcontinent [111]. However, its use is limited due to gastrointestinal, hepatic, renal toxicity, and teratogenic effects. Moreover, the efficiency of miltefosine is rather low in the new world, which may be due to natural resistance in the patients in this region.

Imidazoles and triazoles (ketoconazole, fluconazole, and itraconazole) are antifungal drugs mainly used against CL in the new world. They both act as inhibitors of lanosterol 14α-demethylase in *Leishmania* parasites [112]. Triazoles are metabolized more slowly, interfere less with the synthesis of human sterols, and are, therefore, less toxic than imidazoles [113].

### 7.2. Lipid Status as Prognostic/Diagnostic Biomarker for Leishmaniasis

Although not yet fully understood, nutrition may have a strong influence on the course and severity of leishmaniases. With respect to lipid supply, it was shown that in children with VL and suffering from undernutrition, the development of the disease is favored by a low-fat mass and the disease itself leads to a loss of fat mass [114]. It is now admitted that lipids and lipoproteins play an important role in host defense as well as in the infectivity of trypanosomatids.

Host cholesterol is widely recognized as a key lipid player in *Leishmania* infection [115]. It has been demonstrated that host cell cholesterol is mandatory for the binding, internalization, and development of *Leishmania* into macrophages [7,116] and required for the biogenesis of the parasitophorous vacuole [9,117]. Despite the lack of cholesterol synthesis enzymes in the parasite *Leishmania*, traces of cholesterol have been detected in promastigotes likely reflecting uptake from their environment. An increase in cholesterol content at the expense of ergosterol, the major sterol in *Leishmania* parasites, has been reported in *L. infantum* promastigotes during metacyclogenesis [14]. We previously showed a strong remodeling of sterols during the intramacrophagic transformation of the *L. donovani* and *L. infantum* promastigotes into the amastigote stage, with a marked enrichment in cholesterol and loss of ergosterol [35]. Altogether these observations suggest that macrophage cholesterol is a helping player in *Leishmania* infection.

Increased synthesis of TG and formation of LB were reported in macrophages upon infection with *L. major*, *L. infantum,* or *L. donovani* [118,119,120], and LB of macrophage origin were recovered in the parasitophorous vacuole [118,121]. The formation of LB was also observed in metacyclic *L. infantum* and *L. braziliensis* promastigotes and these organelles were recovered in the parasitophorous vacuole after macrophage infection. Regardless of macrophage or parasite origin, LB have recently emerged as important modulators of *Leishmania* pathogenicity [95,99,119].

With respect to the clinic, it was reported that VL patients had lower LDL, HDL, and total cholesterol levels compared to controls [122,123]. The reduction in serum cholesterol correlates with a high parasite load [124,125]. In mouse models (mice fed a high-cholesterol diet or Apo E-deficient mice), it was demonstrated that high circulating cholesterol levels exert protective effects against *Leishmania* infection [126]. In normocholesterolemic conditions, *L. donovani* infection induces the cholesterol depletion of macrophage membranes, which disorganizes lipid rafts and impairs host cell defense. It was shown that cholesterol replenishment via systemic liposomal cholesterol administration offers protection in hamsters infected with *L. donovani* [114,127]. Liposomal cholesterol delivery would help the host to outwit the *Leishmania* parasite by maintaining a high membrane cholesterol level and activating macrophage immune function [128].

Hypertriglyceridemia has been proposed as a prognostic/diagnostic marker in VL. Patients with VL exhibit increased TG levels at diagnosis that return to normal after VL resolution [122]. Serum TG levels were found to be significantly higher in VL than in control subjects and correlate with the severity of the disease [129,130].

Both hypocholesterolemia and hypertriglyceridemia were measured in pediatric VL patients [131]. Low HDL and elevated TG levels in patients with a mutation of lipoprotein lipase and PPAR alpha genes have been proposed as risk factors for the development of VL [131]. Feeding mice a high-sugar/high-fat diet was shown to increase parasite burden in both the liver and spleen after infection with *L. infantum*/*chagasi* [132]. High TG and low HDL levels were also measured in dogs infected with *L. infantum* [133,134].

It is noteworthy that the levels of cholesterol and triglycerides that impact *Leishmania* infectivity can both be modulated by FAs. Hypothetic mechanisms to explore would be that FAs may increase cholesterol esterification, therefore, reducing free cholesterol and limiting host cell defense. In addition, FAs may modulate TG content through synthesis/esterification/hydrolysis pathways, therefore, interfering with the formation of lipid bodies.

## 8. Conclusions and Perspectives

So far, mainly pharmacological approaches to target specific lipid enzymatic pathways in *Leishmania* parasites, especially involved in PL and sterol biosynthesis and myristoylation, have been used for the development of antileishmanial drugs. As concluding perspectives, we would like to highlight the potential of FAs and PUFAs to modulate parasite or host cell lipid status and consequently parasite/host cell interactions and parasite pathogenicity.

From our data and data from others cited in this review, saturated FA myristate as well as several PUFAs including LA, AA for the n-6 series, and ALA and DHA for the n-3 series appear as FAs of highest interest. Not only do these FAs exhibit changes associated with infection stages, drug sensitivity, or resistance but they are also involved in *Leishmania* metabolism and/or infectivity.

Developing nutritional strategies may thus be worthwhile. Interestingly, Saini et al. [135] reported that serum LA levels were decreased in patients with VL. Macrophage supplementation with LA, either preventively or postinfection, reduced the parasite load in infected macrophages. They suggest that the regular consumption of LA-rich oils in endemic regions may be a valuable strategy to control leishmaniases. In another model of parasite/host cell interaction (i.e., the freshwater crustacean *Daphnia magna* and its parasite *Pasteuria ramosa*), Scholtz et al. [136] showed that a PUFA-enriched diet or specific AA and EPA supplementation significantly reduced the likelihood of infection.

These PUFAs, especially LA, AA, and DHA, have been described as precursors of oxylipins in several *Leishmania* species and infected macrophages. Among them, PGE2, PGF2α, HETE, LTB4, proresolving mediators, and their precursors HDoHE, have been proposed as being involved in *Leishmania* infection. Only a few studies on the oxygen metabolism of PUFAs have been published to date and the characterization of the enzymes has not yet been carried out. Furthermore, the biological activities of specific PUFA metabolites issued from parasites are not well described and deserve further investigation. These lipid mediators certainly open a new and promising way to better understand the role of PUFAs in host cell/parasite interactions and bring them to the fore in therapeutic strategies.

## Figures and Tables

**Table 1:** Comparative FA composition of total lipids in various *Leishmania* species. Nine *Leishmania* strains were isolated from patients in Tunisia including five *L. infantum* strains (A to E) with visceral or cutaneous symptoms and canine leishmaniasis, two *L. tropica* (F,G), and two *L. major* (H,I) strains. FA composition was determined by GC analysis. Data are expressed as mole percent and as means ± SD of four independent determinations. ^a^ indicates significant differences compared to other strains by ANOVA. SFAs, saturated fatty acids; MUFAs, monounsaturated fatty acids.

	*L. infantum*					*L. tropica*		*L. major*	
	MON-1				MON-24	MON-8		MON-25	
	VL			CanL	CL	CL		CL	
	A	B	C	D	E	F	G	H	I
**14:0**	**1.8 ± 0.1**	**2.1 ± 0.1**	**2.1 ± 0.6**	**2.6 ± 0.3**	**2.1 ± 0.1**	**1.9 ± 0.2**	**1.7 ± 0.4**	**6.2 ± 0.9** **^a^**	**6.1 ± 1.0** **^a^**
**16:0**	5.2 ± 1.9	6.4 ± 1.2	5.7 ± 1.2	5.8 ± 0.6	7.2 ± 0.9	5.9 ± 2.9	6.7 ± 0.4	7.0 ± 0.7	6.6 ± 0.6
**18:0**	20.0 ± 1.5	20.1 ± 3.9	21.5 ± 0.9	21.8 ± 1.5	25.3 ± 1.6	17.9 ± 1.8	21.6 ± 4.1	18.9 ± 0.6	18.8 ± 1.7
**20:0**	0.5 ± 0.1	0.8 ± 0.1	0.6 ± 0.1	0.7 ± 0.1	0.4 ± 0.1	1.2 ± 0.3	1.9 ± 0.1	0.4 ± 0.1	0.4 ± 0.4
**SFAs**	27.5	29.3	29.9	30.9	35.0	26.9	31.9	32.5	31.9
**16:1n-9**	nd	nd	nd	nd	nd	nd	nd	0.3 ± 0.1	0.3 ± 0.1
**16:1n-7**	0.5 ± 0.1	0.5 ± 0.4	0.7 ± 0.2	0.6 ± 0.2	0.5 ± 0.2	0.5 ± 0.4	0.8 ± 0.1	1.3 ± 0.1 ^a^	1.2 ± 0.1 ^a^
**18:1 n-9**	23.4 ± 3.3	15.3 ± 2.9	21.5 ± 1.5	15.1 ± 0.6	15.8 ± 1.6	20.4 ± 1.2	22.6 ± 1.2	22.6 ± 0.7	22.7 ± 1.0
**18:1n-7**	1.5 ± 0.2	1.7 ± 0.1	1.7 ± 0.1	1.5 ± 0.1	1.8 ± 0.2	1.5 ± 0.2	1.7 ± 0.1	2.4 ± 0.2 ^a^	2.3 ± 0.2 ^a^
**MUFAs**	25.4	17.5	23.9	17.2	18.1	22.4	25.1	26.6	26.5
**18:2n-6**	21.2 ± 1.7	20.8 ± 3.4	19.1 ± 1.1	22.9 ± 1.2	19.5 ± 0.6	26.5 ± 3.4	14.6 ± 4.0	25.7 ± 0.4	27.9 ± 1.8
**18:3n-6**	0.5 ± 0.1	0.5 ± 0.1	0.6 ± 0.1	0.5 ± 0.1	0.7 ± 0.1	0.7 ± 0.1	0.4 ± 0.2	1.3 ± 0.3 ^a^	1.2 ± 0.2 ^a^
**20:2n-6**	1.3 ± 0.1	1.6 ± 0.1	1.2 ± 0.1	1.7 ± 0.1	1.2 ± 0.2	1.0 ± 0.1	1.0 ± 0.1	0.5 ± 0.1 ^a^	0.6 ± 0.1 ^a^
**20:3n-6**	0.9 ± 0.3	1.6 ± 0.1	1.7 ± 0.1	1.4 ± 0.1	0.8 ± 0.3	1.0 ± 0.2	0.7 ± 0.1	1.5 ± 0.3	1.3 ± 0.2
**20:4 n-6**	0.3 ± 0.1	0.5 ± 0.1	0.4 ± 0.1	0.3 ± 0.0	0.3 ± 0.1	1.0 ± 0.2	0.9 ± 0.2	0.6 ± 0.2	0.6 ± 0.1
**Tot n-6**	24.2	25.0	23.0	26.8	22.5	30.1	17.6	29.6	31.6
**18:3n-3**	**11.2 ± 2.3**	**11.1 ± 0.8**	**9.4 ± 0.8**	**11.2 ± 0.7**	**7.6 ± 1.5**	**9.5 ± 1.4**	**10.0 ± 1.9**	**4.5 ± 1.0 ^a^**	**3.9 ± 0.6 ^a^**
**20:3n-3**	1.7 ± 0.3	2.3 ± 0.2	1.4 ± 0.1	2.2 ± 0.2	1.5 ± 0.3	1.0 ± 0.4	1.9 ± 0.3	0.2 ± 0.1 ^a^	0.2 ± 0.1 ^a^
**20:5n-3**	nd	nd	nd	nd	nd	nd	nd	nd	nd
**22:5n-3**	2.1 ± 0.4	2.7 ± 0.1	2.7 ± 0.3	2.1 ± 0.1	5.1 ± 0.9	1.3 ± 0.3	2.5 ± 0.9	2.3 ± 0.4	2.0 ± 0.3
**22:6n-3**	**7.8 ± 0.7**	**12.0 ± 1.0**	**9.5 ± 1.6**	**9.4 ± 0.6**	**10.1 ± 1.0**	**8.6 ± 2.6**	**11.1 ± 1.6**	**4.2 ± 0.3 ^a^**	**3.8 ± 0.5 ^a^**
**Tot n-3**	22.8	28.1	23.0	24.9	24.3	20.4	25.5	11.2	9.9
**n-3/n-6**	0.9	1.1	1.0	0.9	1.1	0.7	1.4	0.4	0.3

## Data Availability

The data presented in this study are available on request from the corresponding author. The data are not publicly available due to privacy restrictions.

## Abbreviations

AA: arachidonic acid, 20:4n-6; ALA: alpha-linolenic acid, 18:3n-3; CFA: cyclopropanated fatty acid; CL: cutaneous leishmaniasis; COX: cyclooxygenase; CYP: cytochrome P450 oxygenase; DHA: docosahexaenoic acid, 22:6n-3; ELO: elongase; EPA: eicosapentaenoic acid, 20:5n-3; FA: fatty acid; GLA: gamma-linolenic acid, 18:3n-6; LB: Lipid Bodies; LDL: low-density lipoprotein; ALA: alpha-linolenic acid, 18:3n-3; LOX: lipoxygenase; LTB4: leukotriene B4; NO: nitric oxide; OA: oleic acid, 18:1n-9; PA: palmitic acid, 16:0; PC: phosphatidylcholine; PE: phosphatidylethanolamine; PGE2: prostaglandin E2; PGF2: prostaglandin F2; PI: phosphatidylinositol; PL: phospholipid; PUFA: polyunsaturated fatty acid; SA: stearic acid, 18:0; TG: triglyceride; VL: visceral leishmaniasis; FAS: fatty acid synthase.

## References

[1] Murray H.W., Berman J.D., Davies C.R., Saravia N.G. (2005). Advances in Leishmaniasis. Lancet.

[2] Kaye P., Scott P. (2011). Leishmaniasis: Complexity at the Host-Pathogen Interface. Nat. Rev. Microbiol..

[3] Akuffo R., Wilson M., Sarfo B., Attram N., Mosore M.-T., Yeboah C., Cruz I., Ruiz-Postigo J.-A., Boakye D., Moreno J. (2021). Prevalence of *Leishmania* Infection in Three Communities of Oti Region, Ghana. PLoS Negl. Trop. Dis..

[4] Alvar J., Vélez I.D., Bern C., Herrero M., Desjeux P., Cano J., Jannin J., den Boer M. (2012). WHO Leishmaniasis Control Team Leishmaniasis Worldwide and Global Estimates of Its Incidence. PLoS ONE.

[5] Petersen C.A., Barr S.C. (2009). Canine Leishmaniasis in North America: Emerging or Newly Recognized?. Vet. Clin. N. Am. Small Anim. Pract..

[6] Podinovskaia M., Descoteaux A. (2015). *Leishmania* and the Macrophage: A Multifaceted Interaction. Future Microbiol..

[7] Kumar G.A., Jafurulla M., Chattopadhyay A. (2016). The Membrane as the Gatekeeper of Infection: Cholesterol in Host-Pathogen Interaction. Chem. Phys. Lipids.

[8] Schaible U.E., Schlesinger P.H., Steinberg T.H., Mangel W.F., Kobayashi T., Russell D.G. (1999). Parasitophorous Vacuoles of *Leishmania mexicana* Acquire Macromolecules from the Host Cell Cytosol via Two Independent Routes. J. Cell Sci..

[9] Semini G., Paape D., Paterou A., Schroeder J., Barrios-Llerena M., Aebischer T. (2017). Changes to Cholesterol Trafficking in Macrophages by *Leishmania* Parasites Infection. Microbiologyopen.

[10] De Macedo-Silva S.T., de Souza W., Rodrigues J.C.F. (2015). Sterol Biosynthesis Pathway as an Alternative for the Anti-Protozoan Parasite Chemotherapy. Curr. Med. Chem..

[11] Besteiro S., Bertrand-Michel J., Lebrun M., Vial H., Dubremetz J.-F. (2008). Lipidomic Analysis of Toxoplasma Gondii Tachyzoites Rhoptries: Further Insights into the Role of Cholesterol. Biochem. J..

[12] Biagiotti M., Dominguez S., Yamout N., Zufferey R. (2017). Lipidomics and Anti-Trypanosomatid Chemotherapy. Clin. Transl. Med..

[13] Dinesh N., Soumya N., Singh S. (2015). Antileishmanial Effect of Mevastatin Is Due to Interference with Sterol Metabolism. Parasitol. Res..

[14] Yao C., Wilson M.E. (2016). Dynamics of Sterol Synthesis during Development of *Leishmania* Spp. Parasites to Their Virulent Form. Parasites Vectors.

[15] McCall L.-I., El Aroussi A., Choi J.Y., Vieira D.F., De Muylder G., Johnston J.B., Chen S., Kellar D., Siqueira-Neto J.L., Roush W.R. (2015). Targeting Ergosterol Biosynthesis in *Leishmania* Donovani: Essentiality of Sterol 14 Alpha-Demethylase. PLoS Negl. Trop. Dis..

[16] Arya R., Dhembla C., Makde R.D., Sundd M., Kundu S. (2021). An Overview of the Fatty Acid Biosynthesis in the Protozoan Parasite *Leishmania* and Its Relevance as a Drug Target against Leishmaniasis. Mol. Biochem. Parasitol..

[17] Kuhls K., Chicharro C., Cañavate C., Cortes S., Campino L., Haralambous C., Soteriadou K., Pratlong F., Dedet J.-P., Mauricio I. (2008). Differentiation and Gene Flow among European Populations of *Leishmania infantum* MON-1. PLoS Negl. Trop. Dis..

[18] Aoun K., Bouratbine A., Harrat Z., Guizani I., Mokni M., Bel Hadj Ali S., Ben Osman A., Belkaïd M., Dellagi K., Ben Ismaïl R. (2000). Epidemiologic and parasitologic data concerning sporadic cutaneous leishmaniasis in northern Tunisia. Bull. Soc. Pathol. Exot..

[19] Kallel K., Pratlong F., Belhadj S., Cherif F., Hammami M., Dedet J.P., Chaker E. (2005). Cutaneous Leishmaniasis in Tunisia: Results of the Iso-Enzymatic Characterization of 71 Strains. Ann. Trop. Med. Parasitol..

[20] Kallel K., Pratlong F., Haouas N., Kaouech E., Belhadj S., Anane S., Dedet J.P., Babba H., Chaker E. (2008). Isoenzymatic Variability of *Leishmania infantum* in Tunisia Concerning 254 Human Strains. Acta Trop..

[21] Scarpini S., Dondi A., Totaro C., Biagi C., Melchionda F., Zama D., Pierantoni L., Gennari M., Campagna C., Prete A. (2022). Visceral Leishmaniasis: Epidemiology, Diagnosis, and Treatment Regimens in Different Geographical Areas with a Focus on Pediatrics. Microorganisms.

[22] Goto H., Lauletta Lindoso J.A. (2012). Cutaneous and Mucocutaneous Leishmaniasis. Infect. Dis. Clin. N. Am..

[23] De Vries H.J.C., Schallig H.D. (2022). Cutaneous Leishmaniasis: A 2022 Updated Narrative Review into Diagnosis and Management Developments. Am. J. Clin. Derm..

[24] Thomaidou E., Horev L., Jotkowitz D., Zamir M., Ingber A., Enk C.D., Molho-Pessach V. (2015). Lymphatic Dissemination in Cutaneous Leishmaniasis Following Local Treatment. Am. J. Trop. Med. Hyg..

[25] Van Henten S., Adriaensen W., Fikre H., Akuffo H., Diro E., Hailu A., Van der Auwera G., van Griensven J. (2018). Cutaneous Leishmaniasis Due to *Leishmania aethiopica*. EClinicalMedicine.

[26] Strazzulla A., Cocuzza S., Pinzone M.R., Postorino M.C., Cosentino S., Serra A., Cacopardo B., Nunnari G. (2013). Mucosal Leishmaniasis: An Underestimated Presentation of a Neglected Disease. Biomed. Res. Int..

[27] Das V.N.R., Siddiqui N.A., Pandey K., Lal C.S., Sinha S.K., Bimal S., Topno R.K., Singh S.K., Kumar S., Das P. (2019). The Usefulness of Trained Field Workers in Diagnosis of Post-Kala-Azar Dermal Leishmaniasis (PKDL) and Clinico-Epidemiological Profile in Highly Endemic Areas of Bihar. Trans. R. Soc. Trop. Med. Hyg..

[28] Ait Maatallah I., Akarid K., Lemrani M. (2022). Tissue Tropism: Is It an Intrinsic Characteristic of *Leishmania* Species?. Acta Trop..

[29] Beach D.H., Holz G.G., Anekwe G.E. (1979). Lipids of *Leishmania* Promastigotes. J. Parasitol..

[30] Wassef M.K., Fioretti T.B., Dwyer D.M. (1985). Lipid Analyses of Isolated Surface Membranes of *Leishmania donovani* Promastigotes. Lipids.

[31] Adosraku R.K., Anderson M.M., Anderson G.J., Choi G., Croft S.L., Yardley V., Phillipson J.D., Gibbons W.A. (1993). Proton NMR Lipid Profile of *Leishmania donovani* Promastigotes. Mol. Biochem. Parasitol..

[32] Zheng L., T’Kind R., Decuypere S., von Freyend S.J., Coombs G.H., Watson D.G. (2010). Profiling of Lipids in *Leishmania donovani* Using Hydrophilic Interaction Chromatography in Combination with Fourier Transform Mass Spectrometry. Rapid Commun. Mass Spectrom..

[33] De Azevedo A.F., Dutra J.L., Santos M.L., Santos Dde A., Alves P.B., de Moura T.R., de Almeida R.P., Fernandes M.F., Scher R., Fernandes R.P. (2014). Fatty Acid Profiles in *Leishmania* Spp. Isolates with Natural Resistance to Nitric Oxide and Trivalent Antimony. Parasitol. Res..

[34] Mathur R., Das R.P., Ranjan A., Shaha C. (2015). Elevated Ergosterol Protects *Leishmania* Parasites against Antimony-Generated Stress. FASEB J..

[35] Bouazizi-Ben Messaoud H., Guichard M., Lawton P., Delton I., Azzouz-Maache S. (2017). Changes in Lipid and Fatty Acid Composition During Intramacrophagic Transformation of *Leishmania donovani* Complex Promastigotes into Amastigotes. Lipids.

[36] Bouabid C., Yamaryo-Botté Y., Rabhi S., Bichiou H., Hkimi C., Bouglita W., Chaouach M., Eddaikra N., Ghedira K., Guizani-Tabbane L. (2022). Fatty Acid Profiles of *Leishmania major* Derived from Human and Rodent Hosts in Endemic Cutaneous Leishmaniasis Areas of Tunisia and Algeria. Pathogens.

[37] Oyola S.O., Evans K.J., Smith T.K., Smith B.A., Hilley J.D., Mottram J.C., Kaye P.M., Smith D.F. (2012). Functional Analysis of *Leishmania* Cyclopropane Fatty Acid Synthetase. PLoS ONE.

[38] Hsu F.-F., Kuhlmann F.M., Turk J., Beverley S.M. (2014). Multiple-Stage Linear Ion-Trap with High Resolution Mass Spectrometry towards Complete Structural Characterization of Phosphatidylethanolamines Containing Cyclopropane Fatty Acyl Chain in *Leishmania infantum*. J. Mass Spectrom..

[39] Xu W., Mukherjee S., Ning Y., Hsu F.-F., Zhang K. (2018). Cyclopropane Fatty Acid Synthesis Affects Cell Shape and Acid Resistance in *Leishmania mexicana*. Int. J. Parasitol..

[40] Rakotomanga M., Saint-Pierre-Chazalet M., Loiseau P.M. (2005). Alteration of Fatty Acid and Sterol Metabolism in Miltefosine-Resistant *Leishmania donovani* Promastigotes and Consequences for Drug-Membrane Interactions. Antimicrob. Agents Chemother..

[41] Mbongo N., Loiseau P.M., Billion M.A., Robert-Gero M. (1998). Mechanism of Amphotericin B Resistance in *Leishmania donovani* Promastigotes. Antimicrob. Agents Chemother..

[42] t’Kindt R., Scheltema R.A., Jankevics A., Brunker K., Rijal S., Dujardin J.-C., Breitling R., Watson D.G., Coombs G.H., Decuypere S. (2010). Metabolomics to Unveil and Understand Phenotypic Diversity between Pathogen Populations. PLoS Negl. Trop. Dis..

[43] Barratt G., Saint-Pierre-Chazalet M., Loiseau P.M. (2009). Cellular Transport and Lipid Interactions of Miltefosine. Curr. Drug. Metab..

[44] Fernandez-Prada C., Vincent I.M., Brotherton M.-C., Roberts M., Roy G., Rivas L., Leprohon P., Smith T.K., Ouellette M. (2016). Different Mutations in a P-Type ATPase Transporter in *Leishmania* Parasites Are Associated with Cross-Resistance to Two Leading Drugs by Distinct Mechanisms. PLoS Negl. Trop. Dis..

[45] Imbert L., Gaudin M., Libong D., Touboul D., Abreu S., Loiseau P.M., Laprévote O., Chaminade P. (2012). Comparison of Electrospray Ionization, Atmospheric Pressure Chemical Ionization and Atmospheric Pressure Photoionization for a Lipidomic Analysis of *Leishmania donovani*. J. Chromatogr. A.

[46] Gutierrez Guarnizo S.A., Tikhonova E.B., Zabet-Moghaddam M., Zhang K., Muskus C., Karamyshev A.L., Karamysheva Z.N. (2021). Drug-Induced Lipid Remodeling in *Leishmania* Parasites. Microorganisms.

[47] Uttaro A.D. (2014). Acquisition and Biosynthesis of Saturated and Unsaturated Fatty Acids by Trypanosomatids. Mol. Biochem. Parasit..

[48] Parreira de Aquino G., Mendes Gomes M.A., Köpke Salinas R., Laranjeira-Silva M.F. (2021). Lipid and Fatty Acid Metabolism in Trypanosomatids. Microb. Cell..

[49] Lee S.H., Stephens J.L., Englund P.T. (2007). A Fatty-Acid Synthesis Mechanism Specialized for Parasitism. Nat. Rev. Microbiol..

[50] Alloatti A., Uttaro A.D. (2011). Highly Specific Methyl-End Fatty-Acid Desaturases of Trypanosomatids. Mol. Biochem. Parasitol..

[51] Maache M., Azzouz S., Diaz de la Guardia R., Alvarez P., Gil R., de Pablos L.M., Osuna A. (2005). Host Humoral Immune Response to *Leishmania* Lipid-Binding Protein. Parasite Immunol..

[52] Berman J.D., Gallalee J.V., Best J.M., Hill T. (1987). Uptake, Distribution, and Oxidation of Fatty Acids by *Leishmania mexicana* Amastigotes. J. Parasitol..

[53] Leroux M., Bouazizi-Ben Messaoud H., Luquain-Costaz C., Jordheim L.P., Le Faouder P., Gustin M.-P., Aoun K., Lawton P., Azzouz-Maache S., Delton I. (2022). Enriched PUFA Environment of *Leishmania infantum* Promastigotes Promotes the Accumulation of Lipid Mediators and Favors Parasite Infectivity towards J774 Murine Macrophages. Lipids.

[54] De Cicco N.N., Pereira M.G., Correa J.R., Andrade-Neto V.V., Saraiva F.B., Chagas-Lima A.C., Gondim K.C., Torres-Santos E.C., Folly E., Saraiva E.M. (2012). LDL Uptake by *Leishmania* Amazonensis: Involvement of Membrane Lipid Microdomains. Exp. Parasitol..

[55] Andrade-Neto V.V., Cicco N.N.T., Cunha-Junior E.F., Canto-Cavalheiro M.M., Atella G.C., Torres-Santos E.C. (2011). The Pharmacological Inhibition of Sterol Biosynthesis in *Leishmania* Is Counteracted by Enhancement of LDL Endocytosis. Acta Trop..

[56] Coppens I., Courtoy P.J. (2000). The Adaptative Mechanisms of Trypanosoma Brucei for Sterol Homeostasis in Its Different Life-Cycle Environments. Annu. Rev. Microbiol..

[57] Brannigan J.A., Smith B.A., Yu Z., Brzozowski A.M., Hodgkinson M.R., Maroof A., Price H.P., Meier F., Leatherbarrow R.J., Tate E.W. (2010). N-Myristoyltransferase from *Leishmania*
*donovani*: Structural and Functional Characterisation of a Potential Drug Target for Visceral Leishmaniasis. J. Mol. Biol..

[58] Price H.P., Menon M.R., Panethymitaki C., Goulding D., McKean P.G., Smith D.F. (2003). Myristoyl-CoA:Protein N-Myristoyltransferase, an Essential Enzyme and Potential Drug Target in Kinetoplastid Parasites. J. Biol. Chem..

[59] Goldston A.M., Sharma A.I., Paul K.S., Engman D.M. (2014). Acylation in Trypanosomatids: An Essential Process and Potential Drug Target. Trends Parasitol..

[60] Wright M.H., Paape D., Storck E.M., Serwa R.A., Smith D.F., Tate E.W. (2015). Global Analysis of Protein N-Myristoylation and Exploration of N-Myristoyltransferase as a Drug Target in the Neglected Human Pathogen *Leishmania donovani*. Chem. Biol..

[61] Paape D., Prendergast C.T., Price H.P., Doehl J.S.P., Smith D.F. (2020). Genetic Validation of *Leishmania* Genes Essential for Amastigote Survival in Vivo Using N-Myristoyltransferase as a Model. Parasites Vectors.

[62] Bell A.S., Yu Z., Hutton J.A., Wright M.H., Brannigan J.A., Paape D., Roberts S.M., Sutherell C.L., Ritzefeld M., Wilkinson A.J. (2020). Novel Thienopyrimidine Inhibitors of *Leishmania* N-Myristoyltransferase with On-Target Activity in Intracellular Amastigotes. J. Med. Chem..

[63] Corpas-Lopez V., Moniz S., Thomas M., Wall R.J., Torrie L.S., Zander-Dinse D., Tinti M., Brand S., Stojanovski L., Manthri S. (2019). Pharmacological Validation of N-Myristoyltransferase as a Drug Target in *Leishmania donovani*. ACS Infect. Dis..

[64] Saini S., Rai A.K. (2020). Linoleic Acid Inhibits the Release of *Leishmania donovani* Derived Microvesicles and Decreases Its Survival in Macrophages. Front. Cell. Infect. Microbiol..

[65] Su L.-J., Zhang J.-H., Gomez H., Murugan R., Hong X., Xu D., Jiang F., Peng Z.-Y. (2019). Reactive Oxygen Species-Induced Lipid Peroxidation in Apoptosis, Autophagy, and Ferroptosis. Oxid. Med. Cell. Longev..

[66] Reverte M., Snäkä T., Fasel N. (2022). The Dangerous *Liaisons* in the Oxidative Stress Response to *Leishmania* Infection. Pathogens.

[67] Carneiro P.P., Conceição J., Macedo M., Magalhães V., Carvalho E.M., Bacellar O. (2016). The Role of Nitric Oxide and Reactive Oxygen Species in the Killing of *Leishmania braziliensis* by Monocytes from Patients with Cutaneous Leishmaniasis. PLoS ONE.

[68] Deschacht M., Van Assche T., Hendrickx S., Bult H., Maes L., Cos P. (2012). Role of Oxidative Stress and Apoptosis in the Cellular Response of Murine Macrophages upon *Leishmania* Infection. Parasitology.

[69] Gantt K.R., Goldman T.L., McCormick M.L., Miller M.A., Jeronimo S.M., Nascimento E.T., Britigan B.E., Wilson M.E. (2001). Oxidative Responses of Human and Murine Macrophages during Phagocytosis of *Leishmania* Chagasi. J. Immunol..

[70] Roma E.H., Macedo J.P., Goes G.R., Gonçalves J.L., De Castro W., Cisalpino D., Vieira L.Q. (2016). Impact of Reactive Oxygen Species (ROS) on the Control of Parasite Loads and Inflammation in *Leishmania*
*amazonensis* Infection. Parasites Vectors.

[71] Andrade Y.M.F.d.S., de Castro M.V., Tavares V.d.S., Souza R.d.S.O., Faccioli L.H., Lima J.B., Sorgi C.A., Borges V.d.M., Araújo-Santos T. (2022). Polyunsaturated Fatty Acids Alter the Formation of Lipid Droplets and Eicosanoid Production in *Leishmania* Promatigotes. bioRxiv.

[72] Dyall S.C., Balas L., Bazan N.G., Brenna J.T., Chiang N., da Costa Souza F., Dalli J., Durand T., Galano J.-M., Lein P.J. (2022). Polyunsaturated Fatty Acids and Fatty Acid-Derived Lipid Mediators: Recent Advances in the Understanding of Their Biosynthesis, Structures, and Functions. Prog. Lipid Res..

[73] Christie W.W., Harwood J.L. (2020). Oxidation of Polyunsaturated Fatty Acids to Produce Lipid Mediators. Essays Biochem..

[74] Ahmed O.S., Galano J.-M., Pavlickova T., Revol-Cavalier J., Vigor C., Lee J.C.-Y., Oger C., Durand T. (2020). Moving Forward with Isoprostanes, Neuroprostanes and Phytoprostanes: Where Are We Now?. Essays Biochem..

[75] Panis C., Mazzuco T.L., Costa C.Z.F., Victorino V.J., Tatakihara V.L.H., Yamauchi L.M., Yamada-Ogatta S.F., Cecchini R., Rizzo L.V., Pinge-Filho P. (2011). Trypanosoma Cruzi: Effect of the Absence of 5-Lipoxygenase (5-LO)-Derived Leukotrienes on Levels of Cytokines, Nitric Oxide and INOS Expression in Cardiac Tissue in the Acute Phase of Infection in Mice. Exp. Parasitol..

[76] Panis C., Victorino V.J., Tatakihara V.L.H., Cecchini R., Rizzo L.V., Yamauchi L.M., Yamada-Ogatta S.F., Martins-Pinge M.C., Pinge-Filho P. (2019). Differences in CNOS/INOS Activity during Resistance to Trypanosoma Cruzi Infection in 5-Lipoxygenase Knockout Mice. Mediat. Inflamm..

[77] Saini S., Singh B., Prakash S., Kumari S., Kureel A.K., Dube A., Sahasrabuddhe A.A., Rai A.K. (2020). Parasitic Load Determination by Differential Expressions of 5-Lipoxygenase and PGE2 Synthases in Visceral Leishmaniasis. Prostaglandins Other Lipid Mediat..

[78] Sacramento L.A., Cunha F.Q., de Almeida R.P., da Silva J.S., Carregaro V. (2014). Protective Role of 5-Lipoxigenase during *Leishmania infantum* Infection Is Associated with Th17 Subset. Biomed. Res. Int..

[79] Plagge M., Laskay T. (2017). Early Production of the Neutrophil-Derived Lipid Mediators LTB4 and LXA4 Is Modulated by Intracellular Infection with *Leishmania major*. Biomed. Res. Int..

[80] Lefèvre L., Lugo-Villarino G., Meunier E., Valentin A., Olagnier D., Authier H., Duval C., Dardenne C., Bernad J., Lemesre J.L. (2013). The C-Type Lectin Receptors Dectin-1, MR, and SIGNR3 Contribute Both Positively and Negatively to the Macrophage Response to *Leishmania infantum*. Immunity.

[81] Serezani C.H., Perrela J.H., Russo M., Peters-Golden M., Jancar S. (2006). Leukotrienes Are Essential for the Control of *Leishmania amazonensis* Infection and Contribute to Strain Variation in Susceptibility. J. Immunol..

[82] Tavares N., Afonso L., Suarez M., Ampuero M., Prates D.B., Araújo-Santos T., Barral-Netto M., DosReis G.A., Borges V.M., Brodskyn C. (2016). Degranulating Neutrophils Promote Leukotriene B4 Production by Infected Macrophages To Kill *Leishmania amazonensis* Parasites. J. Immunol..

[83] Tavares N.M., Araújo-Santos T., Afonso L., Nogueira P.M., Lopes U.G., Soares R.P., Bozza P.T., Bandeira-Melo C., Borges V.M., Brodskyn C. (2014). Understanding the Mechanisms Controlling *Leishmania amazonensis* Infection in Vitro: The Role of LTB4 Derived from Human Neutrophils. J. Infect. Dis..

[84] Chaves M., Savio L.E., Coutinho-Silva R. (2022). Purinergic Signaling: A New Front-Line Determinant of Resistance and Susceptibility in Leishmaniasis. Biomed. J..

[85] Bhattacharjee A., Majumder S., Das S., Ghosh S., Biswas S., Majumdar S. (2016). *Leishmania donovani*-Induced Prostaglandin E2 Generation Is Critically Dependent on Host Toll-Like Receptor 2-Cytosolic Phospholipase A2 Signaling. Infect. Immun..

[86] Saha A., Biswas A., Srivastav S., Mukherjee M., Das P.K., Ukil A. (2014). Prostaglandin E2 Negatively Regulates the Production of Inflammatory Cytokines/Chemokines and IL-17 in Visceral Leishmaniasis. J. Immunol..

[87] López-Muñoz R.A., Molina-Berríos A., Campos-Estrada C., Abarca-Sanhueza P., Urrutia-Llancaqueo L., Peña-Espinoza M., Maya J.D. (2018). Inflammatory and Pro-Resolving Lipids in Trypanosomatid Infections: A Key to Understanding Parasite Control. Front. Microbiol..

[88] Penke L.R., Sudan R., Sathishkumar S., Saha B. (2013). Prostaglandin E₂ Receptors Have Differential Effects on *Leishmania major* Infection. Parasite Immunol..

[89] Guimarães E.T., Santos L.A., Ribeiro dos Santos R., Teixeira M.M., dos Santos W.L.C., Soares M.B.P. (2006). Role of Interleukin-4 and Prostaglandin E2 in *Leishmania amazonensis* Infection of BALB/c Mice. Microbes Infect..

[90] Lima J.B., Araújo-Santos T., Lázaro-Souza M., Carneiro A.B., Ibraim I.C., Jesus-Santos F.H., Luz N.F., Pontes S.d.M., Entringer P.F., Descoteaux A. (2017). *Leishmania infantum* Lipophosphoglycan Induced-Prostaglandin E2 Production in Association with PPAR-γ Expression via Activation of Toll like Receptors-1 and 2. Sci. Rep..

[91] Gregory D.J., Sladek R., Olivier M., Matlashewski G. (2008). Comparison of the Effects of *Leishmania major* or *Leishmania donovani* Infection on Macrophage Gene Expression. Infect. Immun..

[92] Colas R.A., Ashton A.W., Mukherjee S., Dalli J., Akide-Ndunge O.B., Huang H., Desruisseaux M.S., Guan F., Jelicks L.A., Matos Dos Santos F. (2018). Trypanosoma Cruzi Produces the Specialized Proresolving Mediators Resolvin D1, Resolvin D5, and Resolvin E2. Infect. Immun..

[93] Machado F.S., Mukherjee S., Weiss L.M., Tanowitz H.B., Ashton A.W. (2011). Bioactive Lipids in Trypanosoma Cruzi Infection. Adv. Parasitol..

[94] Paloque L., Perez-Berezo T., Abot A., Dalloux-Chioccioli J., Bourgeade-Delmas S., Le Faouder P., Pujo J., Teste M.-A., François J.-M., Schebb N.H. (2019). Polyunsaturated Fatty Acid Metabolites: Biosynthesis in *Leishmania* and Role in Parasite/Host Interaction. J. Lipid Res..

[95] Tavares V.d.S., de Castro M.V., Souza R.d.S.O., Gonçalves I.K.A., Lima J.B., Borges V.d.M., Araújo-Santos T. (2022). Lipid Droplets of Protozoan Parasites: Survival and Pathogenicity. Memórias Do Inst. Oswaldo Cruz.

[96] Niu M., Keller N.P. (2019). Co-Opting Oxylipin Signals in Microbial Disease. Cell. Microbiol..

[97] Kubata B.K., Duszenko M., Kabututu Z., Rawer M., Szallies A., Fujimori K., Inui T., Nozaki T., Yamashita K., Horii T. (2000). Identification of a Novel Prostaglandin f(2alpha) Synthase in Trypanosoma Brucei. J. Exp. Med..

[98] Díaz-Viraqué F., Chiribao M.L., Trochine A., González-Herrera F., Castillo C., Liempi A., Kemmerling U., Maya J.D., Robello C. (2018). Old Yellow Enzyme from Trypanosoma Cruzi Exhibits In Vivo Prostaglandin F2α Synthase Activity and Has a Key Role in Parasite Infection and Drug Susceptibility. Front. Immunol..

[99] Araujo-Santos T., Prates D.B., Franca-Costa J., Luz N.F., Andrade B.B., Miranda J.C., Brodskyn C.I., Barral A., Bozza P.T., Borges V.M. (2014). Prostaglandin E2/Leukotriene B4 Balance Induced by Lutzomyia Longipalpis Saliva Favors *Leishmania infantum* Infection. Parasites Vectors.

[100] Alves-Ferreira E.V.C., Ferreira T.R., Walrad P., Kaye P.M., Cruz A.K. (2020). *Leishmania braziliensis* Prostaglandin F2α Synthase Impacts Host Infection. Parasit Vectors.

[101] Estrada-Figueroa L.A., Díaz-Gandarilla J.A., Hernández-Ramírez V.I., Arrieta-González M.M., Osorio-Trujillo C., Rosales-Encina J.L., Toledo-Leyva A., Talamás-Rohana P. (2018). *Leishmania mexicana* Gp63 Is the Enzyme Responsible for Cyclooxygenase (COX) Activity in This Parasitic Protozoa. Biochimie.

[102] Kabututu Z., Martin S.K., Nozaki T., Kawazu S., Okada T., Munday C.J., Duszenko M., Lazarus M., Thuita L.W., Urade Y. (2003). Prostaglandin Production from Arachidonic Acid and Evidence for a 9,11-Endoperoxide Prostaglandin H2 Reductase in *Leishmania*. Int. J. Parasitol..

[103] Kubata B.K., Duszenko M., Martin K.S., Urade Y. (2007). Molecular Basis for Prostaglandin Production in Hosts and Parasites. Trends Parasitol..

[104] Malta-Santos H., Andrade B.B., Zanette D.L., Costa J.M., Bozza P.T., Bandeira-Melo C., Barral A., França-Costa J., Borges V.M. (2017). Resolvin D1 Drives Establishment of *Leishmania amazonensis* Infection. Sci. Rep..

[105] Esmaeeli S., Hoseinirad S.M., Rajabian M., Taheri A.R., Berenji F., Hashemy S.I. (2019). Evaluation of the Oxidant-Antioxidant Balance, Isoprostane and Quantitative CRP in Patients with Cutaneous Leishmaniasis. Microb. Pathog..

[106] Roberts A.J., Dunne J., Scullion P., Norval S., Fairlamb A.H. (2018). A Role for Trypanosomatid Aldo-Keto Reductases in Methylglyoxal, Prostaglandin and Isoprostane Metabolism. Biochem. J..

[107] Wyllie S., Vickers T.J., Fairlamb A.H. (2008). Roles of Trypanothione S-Transferase and Tryparedoxin Peroxidase in Resistance to Antimonials. Antimicrob. Agents Chemother..

[108] Pavli A., Maltezou H.C. (2010). Leishmaniasis, an Emerging Infection in Travelers. Int. J. Infect. Dis..

[109] Polonio T., Efferth T. (2008). Leishmaniasis: Drug Resistance and Natural Products (Review). Int. J. Mol. Med..

[110] Ríos-Marco P., Marco C., Gálvez X., Jiménez-López J.M., Carrasco M.P. (2017). Alkylphospholipids: An Update on Molecular Mechanisms and Clinical Relevance. Biochim. Biophys. Acta (BBA)-Biomembr..

[111] Carnielli J.B.T., Crouch K., Forrester S., Silva V.C., Carvalho S.F.G., Damasceno J.D., Brown E., Dickens N.J., Costa D.L., Costa C.H.N. (2018). A *Leishmania infantum* Genetic Marker Associated with Miltefosine Treatment Failure for Visceral Leishmaniasis. EBioMedicine.

[112] Emami S., Tavangar P., Keighobadi M. (2017). An Overview of Azoles Targeting Sterol 14α-Demethylase for Antileishmanial Therapy. Eur. J. Med. Chem..

[113] Mitropoulos P., Konidas P., Durkin-Konidas M. (2010). New World Cutaneous Leishmaniasis: Updated Review of Current and Future Diagnosis and Treatment. J. Am. Acad. Derm..

[114] Harrison L.H., Naidu T.G., Drew J.S., de Alencar J.E., Pearson R.D. (1986). Reciprocal Relationships between Undernutrition and the Parasitic Disease Visceral Leishmaniasis. Rev. Infect. Dis..

[115] Andrade-Neto V.V., Manso P.P.d.A., Pereira M.G., de Cicco N.N.T., Atella G.C., Pelajo-Machado M., Menna-Barreto R.F.S., Torres-Santos E.C. (2022). Host Cholesterol Influences the Activity of Sterol Biosynthesis Inhibitors in *Leishmania amazonensis*. Memórias Do Inst. Oswaldo Cruz.

[116] Pucadyil T.J., Chattopadhyay A. (2007). Cholesterol: A Potential Therapeutic Target in *Leishmania* Infection?. Trends Parasitol..

[117] Pessoa C.C., Reis L.C., Ramos-Sanchez E.M., Orikaza C.M., Cortez C., de Castro Levatti E.V., Badaró A.C.B., Yamamoto J.U.d.S., D’Almeida V., Goto H. (2019). ATP6V0d2 Controls *Leishmania* Parasitophorous Vacuole Biogenesis via Cholesterol Homeostasis. PLoS Pathog..

[118] Rodríguez N.E., Lockard R.D., Turcotte E.A., Araújo-Santos T., Bozza P.T., Borges V.M., Wilson M.E. (2017). Lipid Bodies Accumulation in *Leishmania infantum*-Infected C57BL/6 Macrophages. Parasite Immunol..

[119] Banerjee S., Bose D., Das S., Chatterjee N., Mishra S., Das Saha K. (2022). *Leishmania donovani* Infection Induce Extracellular Signal-Regulated Kinase ½ (ERK½) Mediated Lipid Droplet Generation in Macrophages. Mol. Immunol..

[120] Rabhi I., Rabhi S., Ben-Othman R., Rasche A., Daskalaki A., Trentin B., Piquemal D., Regnault B., Descoteaux A., Guizani-Tabbane L. (2012). Transcriptomic Signature of *Leishmania* Infected Mice Macrophages: A Metabolic Point of View. PLoS Negl. Trop. Dis..

[121] Rabhi S., Rabhi I., Trentin B., Piquemal D., Regnault B., Goyard S., Lang T., Descoteaux A., Enninga J., Guizani-Tabbane L. (2016). Lipid Droplet Formation, Their Localization and Dynamics during *Leishmania major* Macrophage Infection. PLoS ONE.

[122] Liberopoulos E.N., Apostolou F., Gazi I.F., Kostara C., Bairaktari E.T., Tselepis A.D., Elisaf M. (2014). Visceral Leishmaniasis Is Associated with Marked Changes in Serum Lipid Profile. Eur. J. Clin. Investig..

[123] Soares N.M., Leal T.F., Fiúza M.C., Reis E.a.G., Souza M.a.L., Dos-Santos W.L., Pontes-de-Carvalho L. (2010). Plasma Lipoproteins in Visceral Leishmaniasis and Their Effect on *Leishmania*-Infected Macrophages. Parasite Immunol..

[124] Lal C.S., Verma N., Rabidas V.N., Ranjan A., Pandey K., Verma R.B., Singh D., Kumar S., Das P. (2010). Total Serum Cholesterol Determination Can Provide Understanding of Parasite Burden in Patients with Visceral Leishmaniasis Infection. Clin. Chim. Acta.

[125] Ghosh J., Lal C.S., Pandey K., Das V.N.R., Das P., Roychoudhury K., Roy S. (2011). Human Visceral Leishmaniasis: Decrease in Serum Cholesterol as a Function of Splenic Parasite Load. Ann. Trop. Med. Parasitol..

[126] Ghosh J., Das S., Guha R., Ghosh D., Naskar K., Das A., Roy S. (2012). Hyperlipidemia Offers Protection against *Leishmania donovani* Infection: Role of Membrane Cholesterol. J. Lipid Res..

[127] Banerjee S., Ghosh J., Sen S., Guha R., Dhar R., Ghosh M., Datta S., Raychaudhury B., Naskar K., Haldar A.K. (2009). Designing Therapies against Experimental Visceral Leishmaniasis by Modulating the Membrane Fluidity of Antigen-Presenting Cells. Infect. Immun..

[128] Ghosh J., Guha R., Das S., Roy S. (2014). Liposomal Cholesterol Delivery Activates the Macrophage Innate Immune Arm to Facilitate Intracellular *Leishmania donovani* Killing. Infect. Immun..

[129] Lal C.S., Verma R.B., Verma N., Siddiqui N.A., Rabidas V.N., Pandey K., Singh D., Kumar S., Paswan R.K., Kumari A. (2016). Hypertriglyceridemia: A Possible Diagnostic Marker of Disease Severity in Visceral Leishmaniasis. Infection.

[130] Varela M.G., de Oliveira Bezerra M., Santana F.V., Gomes M.C., de Jesus Almeida P.R., Silveira da Cruz G., de Melo E.V., de Oliveira Costa P.R., de Oliveira F.A., de Jesus A.R. (2021). Association between Hypertriglyceridemia and Disease Severity in Visceral Leishmaniasis. Am. J. Trop. Med. Hyg..

[131] Carvalho M.D.T., Alonso D.P., Vendrame C.M.V., Costa D.L., Costa C.H.N., Werneck G.L., Ribolla P.E.M., Goto H. (2014). Lipoprotein Lipase and PPAR Alpha Gene Polymorphisms, Increased Very-Low-Density Lipoprotein Levels, and Decreased High-Density Lipoprotein Levels as Risk Markers for the Development of Visceral Leishmaniasis by *Leishmania infantum*. Mediat. Inflamm..

[132] Sarnáglia G.D., Covre L.P., Pereira F.E.L., DE Matos Guedes H.L., Faria A.M.C., Dietze R., Rodrigues R.R., Maioli T.U., Gomes D.C.O. (2016). Diet-Induced Obesity Promotes Systemic Inflammation and Increased Susceptibility to Murine Visceral Leishmaniasis. Parasitology.

[133] Nieto C.G., Barrera R., Habela M.A., Navarrete I., Molina C., Jiménez A., Serrera J.L. (1992). Changes in the Plasma Concentrations of Lipids and Lipoprotein Fractions in Dogs Infected with *Leishmania infantum*. Vet. Parasitol..

[134] Khaleghi Einakchi M., Sedaghat Sharifi N., Khoshnegah J., Heidarpour M. (2018). Canine Visceral Leishmaniosis: The Relationship of Blood Serum Thyroid Hormones, Lipids, and Lipoproteins with Clinical Status. Parasitol. Res..

[135] Saini S., Kottarath S.K., Dinda A.K., Dube A., Sahasrabuddhe A.A., Thakur C.P., Bhat M., Rai A.K. (2020). Preventive as Well as Therapeutic Significances of Linoleic Acid in the Containment of *Leishmania donovani* Infection. Biochimie.

[136] Schlotz N., Ebert D., Martin-Creuzburg D. (2013). Dietary Supply with Polyunsaturated Fatty Acids and Resulting Maternal Effects Influence Host--Parasite Interactions. BMC Ecol..

